# Bilateral Gluteal Augmentation With Hyperdilute Calcium Hydroxylapatite Microspheres Performed Using the Bella Vida Instant Brazilian Butt Lift (BBL)™

**DOI:** 10.7759/cureus.26261

**Published:** 2022-06-23

**Authors:** Iani Silveira, Brigitte Martinez

**Affiliations:** 1 Dermatology, Bella Vida Aesthetics & Wellness, Miami, USA; 2 Nursing, Keiser University, Miami, USA

**Keywords:** biostimulator, non surgical butt lift, minimally invasive, caha, radiesse, gluteal augmentation, butt lift, bbl

## Abstract

For decades, aesthetic medical procedures have sought to improve patient appearance, comfort, and self-confidence. In more recent years, a growing interest in body contouring cosmetic procedures has emerged, in large part due to increased transparency in procedures and improved outcomes with minimally invasive procedures. Notably, several biostimulatory fillers have emerged as a suitable treatment modality due to their relatively low cost, low pain, short downtime, high tunability, and sustained biostimulatory effect. One volumizing biostimulatory filler, Radiesse® (Merz Aesthetics, Frankfurt, Germany), consists of calcium hydroxylapatite microspheres suspended in a carboxymethyl cellulose gel. When injected, Radiesse immediately volumizes the injection site and initiates neocollagenesis and neoelastogenesis. One technique, the Bella Vida Instant Brazilian Butt Lift (BBL)™, is a fast, safe, and minimally invasive alternative to surgical gluteal augmentation that accomplishes morphological remodeling, increases volumization, and improves shape and patient satisfaction. This technical report provides the clinical basis, materials, and methods for implementing the Bella Vida Instant BBL™ in aesthetic practices.

## Introduction

Over the last several years, interest in cosmetic procedures has increased [1]. Notably, minimally or non-invasive cosmetic procedures have grown in popularity due to their minimal pain and recovery time, ease, procedural speed, exceptional biological responses, and positive effect on self-image [2,3]. For minimally invasive body contouring, a variety of energy-based [4-6] and injectable materials [7-10] are commonly utilized. Notably, biostimulatory fillers have the potential to add volume to volumetrically-deficient tissues and concurrently drive endogenous extracellular matrix (ECM) regeneration [11,12]. One biostimulatory filler, calcium hydroxylapatite (CaHA; Radiesse®; Merz Aesthetics, Frankfurt, Germany), drives type I and III collagen and elastin synthesis via mechanical stimulation of native fibroblasts [12-14].

## Technical report

Background

**Figure 1:**
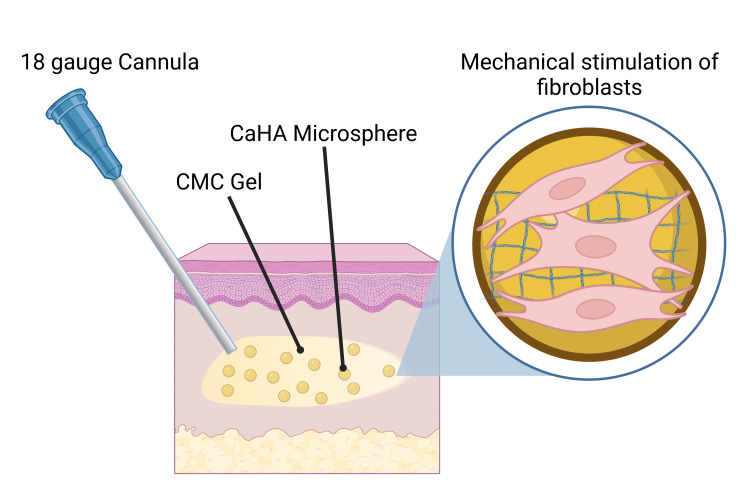
Proposed biostimulatory mechanism of action of CaHA injected subdermally via 18 gauge cannula The carboxymethyl cellulose (CMC) gel immediately volumizes the tissue while the CaHA microspheres mechanically stimulate local fibroblasts for type I and type III collagen and elastin synthesis.

Unlike other biostimulators, CaHA has a volumizing component (carboxymethyl cellulose (CMC) gel; 70% w/v) and a biostimulating component (CaHA microspheres 30% w/v) that, when injected, provides immediate volumization and subsequent biostimulation via cell-material interaction (Figure 1). Therefore, CaHA treatment results in an immediate correction from the CMC gel, which is gradually replaced by endogenous collagen and elastin. In recent years, hyperdilute Radiesse (HDR), defined as CaHA diluted at >1:1, has become a safe, well-tolerated, and morphologically optimal aesthetic treatment for body contouring [9,10,15]. Due to the increase in dilution volume, HDR tends to spread over larger tissue volumes while still driving ECM synthesis. For larger anatomies, HDR treatments may be preferable due to the increased tissue volume undergoing biostimulation.

**Figure 2:**
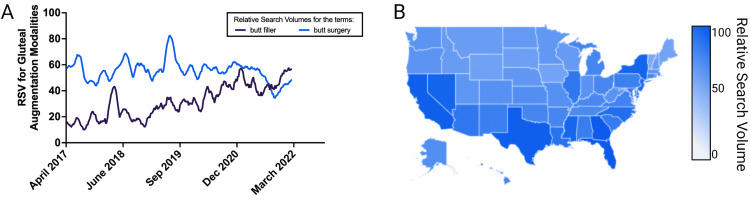
Relative search volume in gluteal augmentation procedures (A) Five-year search interest in the terms 'butt filler' and 'butt surgery' as proxies for interest in minimally invasive and surgical gluteal augmentation procedures in the US. (B) Geographical interest in buttock augmentation demonstrates widespread geospatial interest. Data source for this figure is Google Trends; RSV - relative search volume

Gluteal augmentation may be a treatment modality to augment tissue, treat asymmetry, or increase self-confidence [16]. Gluteal augmentation can be accomplished with surgical implantation, fat transfer, or, more recently, with minimally invasive biostimulatory fillers like CaHA [11,16-19]. Though surgical intervention was largely preferred for many years, minimally invasive treatments have grown in popularity due to their favorable outcomes, fast recovery times, low overhead, and accessibility [2]. To empirically capture the shift in interest from surgical to minimally invasive gluteal augmentation, a five-year retrospective analysis of Google Trends relative search volume (RSV) was conducted, as many Google Trend studies have demonstrated the relationship between internet search volume and procedural participation [20-24]. Our analysis demonstrates a shifting interest from surgical gluteal augmentation ('butt surgery') to injectable augmentation ('butt filler') (Figure 2). In 2021, for the first time, RSV for 'butt filler' surpassed that of 'butt surgery' in the United States (Figure 2A). Additionally, interest in gluteal augmentation is not geographically confined to one region or state, as evident by the states with the five highest RSV coming from a variety of US regions: Florida (100; southeast), Georgia (97; southeast), Texas (94; south-central), California (93; west), and New York (88; northeast) (Figure 2B). 

Objective

Noting this shift in patient preference, many injectors and aesthetic providers have adopted the practice of HDR gluteal augmentation. Though several consensus articles exist suggesting generalized protocols, we report for the first time, bilateral gluteal augmentation with HDR CaHA using the Bella Vida Instant Brazilian butt lift (BBL)™ (BVBBL). In this technical report, we describe the materials, methods, and considerations for optimal aesthetic outcomes and minimal adverse event rates. In addition to material preparation and safety considerations, this technical report outlines protocols for addressing: shelf, hip dips, heart shape, and projection augmentation techniques. BVBBL consists of a general, but customizable procedure using bolus and retrograde placement of HDR injected via cannula, with distinct treatment plans based on the desired buttock shape and expected patient outcomes.

Materials

**Figure 3:**
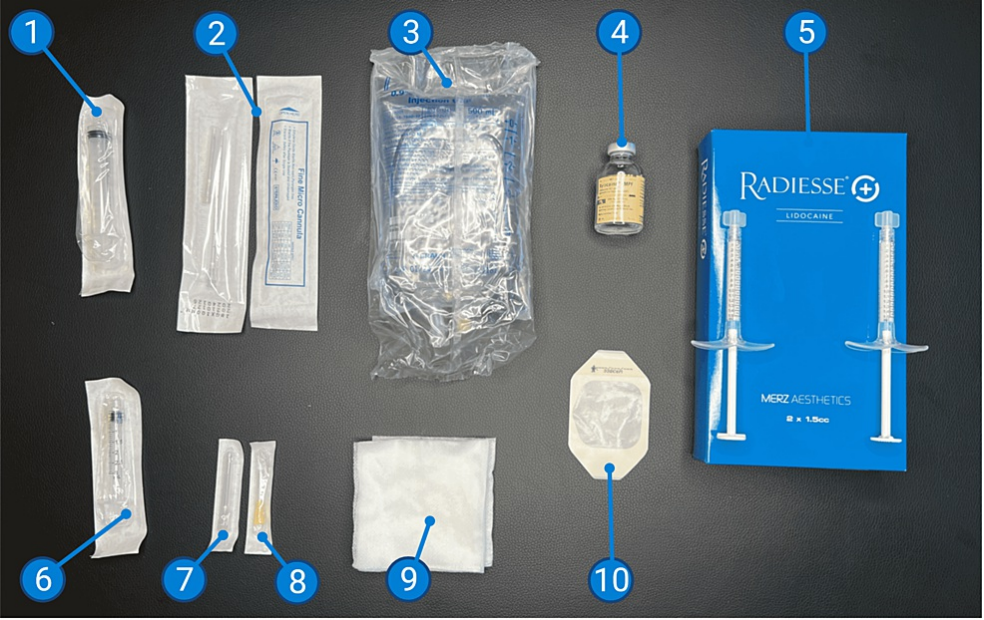
Materials utilized in the Bella Vida Instant BBL™ 1) 10 ml syringe; 2) 18 gauge, 1.5” cannula; 3) 0.9% NaCl saline; 4) lidocaine 2%; 5) CaHA (Radiesse); 6) 3 ml syringe; 7) 18 gauge and 30 gauge needles, respectively; 8) non-woven gauze; 9) Tegaderm™ BBL - Brazilian butt lift

A variety of materials are utilized for this procedure and are outlined in Table 1 and pictured in Figure 3. Injected materials include lidocaine 2% (Xylocaine HCl; 0.9% NaCl saline (B. Braun, Melsungen, Germany)), and CaHA (Radiesse). Materials for injection include 10 ml Luer Lock syringes (McKesson Corporation, Irvine, Texas), 18 and 30 gauge 1.5" needles (McKesson Corporation, Irvine, Texas), 3 ml Luer Lock syringes (McKesson Corporation, Irvine, Texas), and 18 gauge Fine Micro Cannulas (Microsculpt, New York City, New York). Peripheral materials include non-woven sponges (Dynarex Corporation, Orangeburg, New York) and Tegaderm™ (3M™, Saint Paul, Minnesota). The full list of materials for the procedures is listed in Table 1 and pictured in Figure 3.

**Table 1:** Materials required for the Bella Vida BBL™ BBL - Brazilian butt lift

Material	Manufacturer name	City, state	Country
10 ml Luer Lock syringe	McKesson Corporation	Irvine, TX	USA
18 ga, 3.9” Blunt Tip Cannula	Microsculpt	New York City, NY	USA
0.9% NaCL	B. Braun	Melsungen	Germany
Lidocaine 2% (Xylocaine HCl)	Fresenius	Bad Homburg	Germany
CaHA (Radiesse)	Merz Aesthetics	Frankfurt	Germany
3 ml Luer Lock syringe	McKesson Corporation	Irvine, TX	USA
18 ga, 1.5' needle	McKesson Corporation	Irvine, TX	USA
30 ga, 1.5" needle	McKesson Corporation	Irvine, TX	USA
Non-woven Sponges	Dynarex	Orangeburg, NY	USA
Tegaderm	3M	Saint Paul, MN	USA

Preparation

**Figure 4:**
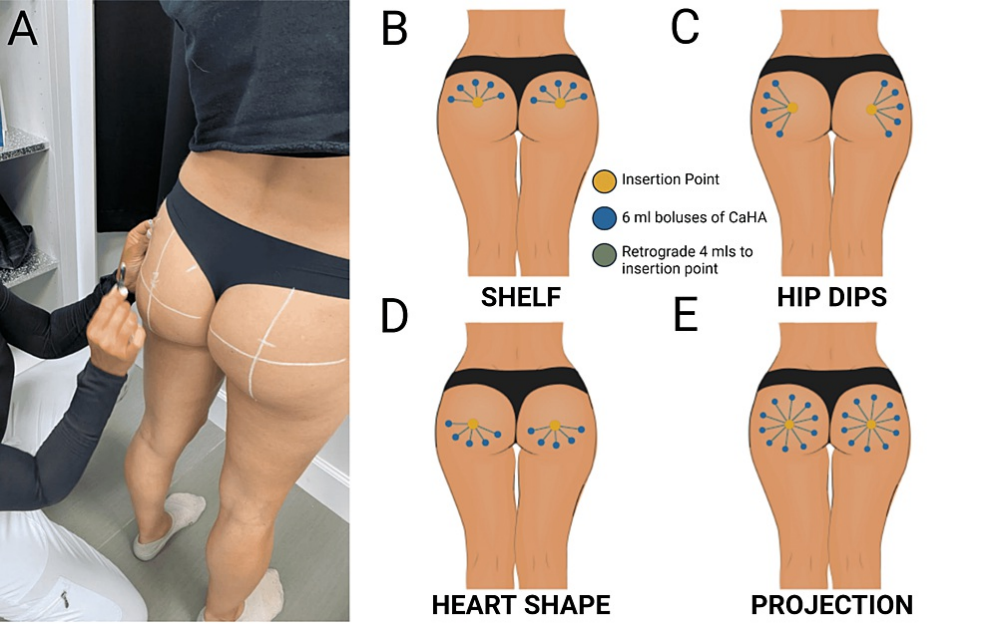
Injection marking and techniques for shape-distinct augmentation (A) Anatomical mapping of injection sites; (B-E) Insertion points (yellow), CaHA bolus locations (blue), and retrograde paths (green) for shelf, hip dips, heart shape, and projection treatments, respectively.

Before augmentation begins, material and patient preparation must occur. First, 8.5 ml of normal saline is drawn into each 10 ml syringe. A Luer Lock transfer adapter is fastened to a 1.5 ml syringe of Radiesse and to the 10 ml saline-filled syringe. Next, the Radiesses is transferred into the 10 ml syringe, creating a 1:6 ratio of CaHA to saline. The rationale behind selecting a 1:6 dilution is largely based on the increased lateral and deep spread of the product [9,10,12,13]. In other words, treating a large volume of tissue (i.e., the buttocks) requires a significant spread of the product to optimize the volume of tissue undergoing biostimulation. Though the per tissue volume biostimulation decreases with increasing dilution, a significant amount of collagen and elastin is stimulated with hyperdilutions of CaHA [13]. Lower dilutions may stimulate more endogenous collagen and elastin, but a larger volume of the product would be required to treat the same tissue volume. After hyperdiluting to a 1:6 dilution, the Radiesse syringe (now empty) is removed and replaced with another 10 ml syringe. The Radiesse and saline solution is passed from syringe to syringe 20 times to facilitate homogenization. CaHA microspheres may remain suspended for up to 20 min in our experience, but agitation to resuspend the product immediately prior to injection is considered the best practice for retaining homogeneity. These steps are repeated until the desired number of syringes are prepared (this varies based on the desired outcome and tissue volume treated). Next, 2 ml of 2% lidocaine with/without epinephrine is drawn into a 3 ml syringe with a 30 ga. needle. At this point, the preparation tray should include the pre-filled syringes of filler (HDR CaHA, 1:6), lidocaine with epinephrine, antiseptic for cleaning, 18 gauge needle to open insertion site, and 18 gauge cannula for insertion of product. Once the preparation area is complete, the provider should ask the patient to stand in an upright position so that mapping lines can be drawn out (Figure 4A). At this point, the provider should know the desired clinical outcome (shape and size) and should map accordingly. There are four distinct treatments for the BVIBBL: shelf, hip dips, heart shape, and projection. The general procedure is similar for each pattern, but a brief pattern-specific outline will be outlined as well.

Procedure

First, each insertion site is cleaned with an antiseptic wipe. Then, using a 3 ml lidocaine syringe, 1 ml of lidocaine is injected at each insertion point. If Radiesse (+) (CaHA with 0.3% integral lidocaine) is used for this procedure, additional lidocaine is not necessary. Next, an 18 ga. needle is inserted into the pre-marked and numbed insertion site location to pilot the insertion site hole. Next, the 18 ga. cannula attached to the 10 ml syringe of HDR CaHA is inserted into the subdermal plane of the glute. It is imperative to ensure that the cannula is in the correct plane. This can be accomplished by moving the cannula side-to-side and observing if there is resistance. If the movement is smooth, it is likely that the cannula is in the correct plane. Next, the cannula is positioned at the desired location according to mapping. Regardless of the pattern selected, each procedure begins by injecting a 6 ml bolus and subsequently retrograding the remaining 4 ml of HDR CaHA, stopping just before the insertion point, so the HDR CaHA does not expel out of the body. After all the HDR CaHA from the first syringe is placed, the cannula is slowly withdrawn until only a small portion of the tip remains inserted. The empty syringe is removed and replaced with the next premixed 10 ml syringe of HDR CaHA. Next, the cannula is reinserted into the same subdermal plane, and the next 6 ml bolus is injected at the marked location, leaving approximately 2 cm between the last 6 ml bolus. The remaining 4 ml of product is then retrograded towards the insertion site. These steps are repeated until the desired tissue area is fully treated. After all bolus-to-retrograde fanning is finished, remove the cannula and gently apply pressure to the insertion point using gauze for up to 3 minutes, or until any leakage has subsided. Finally, apply Tegaderm to the injection site once blood and product have stopped exuding. The following subsections include protocols for shape-specific augmentations.

Shelf

The desired outcome for the shelf pattern is increased superior projection. The insertion point is positioned in the center on the x-axis of each glute and slightly above the midline on the y-axis. Five evenly-spaced boluses are placed along the top of each glute after advancing the cannula superiorly. The remaining 4 ml of HDR CaHA is deposited in retrograde threads towards the insertion point (Figure 4B)

Hip dips

The desired outcome for hip dips is to increase lateral projection yielding a rounder appearance and contributing to a "Coke-bottle shape". Insertion points are positioned in the center of both the x and y axes. Boluses are evenly distributed in a lateral arrangement, with the cannula first advancing towards the outline of the hip dips. The remaining 4 ml of HDR CaHA is deposited by retrograde threads during withdrawal towards the insertion point (Figure 4C). 

Heart shape

To create the bottom-heavy desired outcome for glutes that have too much skin laxity or need more volume for the aesthetically-pleasing "sweet-heart" shape, boluses are laid in the inferior portion of the glute with a similar retrograde as indicated in previous approaches. This area will take about 24 to 48 hours to round out due to increased pressure in the tissue (Figure 4D).

Projection

This procedure is for rounding out and correcting the volumetric and superficial appearance of all surrounding tissue in the glutes. Using the same centrally-located insertion point, 6 ml boluses are placed in a complete circle around the glute, and 4 ml of HDR CaHA is deposited via retrograde deposition. As explained in Figure 4E, this method is to correct all surrounding areas and create volume outward.

Post-procedure care

Healthcare providers (HCPs) should advise patients on the importance of avoiding: the application of pressure to the insertion site (though no compression garments are required), strenuous activity, sun and heat exposure, and submersion of water for a minimum of 48 hours. Vigorous exercising should be avoided for a minimum of 48 hours. Additionally, the Tegaderm should remain in place for 48 hours after the procedure to mitigate the risk of losing product from the insertion site. Oral nonsteroidal anti-inflammatory medications can be administered at the patient's discretion and as needed. Additionally, the use of oral supplements (i.e., minerals, vitamins, and micronutrient supplementation) may help increase aesthetic outcomes.

## Discussion

Though many patients may benefit from the BVIBBL, an ideal patient is generally one who is not suited for surgical gluteal augmentation. This may include patients who desire short recovery periods, do not possess adequate adipose tissue required for a lipotransfer, or are sensitive to anesthetics. On the other hand, an ideal patient may have a relatively low BMI or may want to address the following: deep to superficial cellulite, stretch marks, asymmetries (in volume, shape, or appearance), volume deficits, or skin laxity. To this end, HDR CaHA is a tool to personalize patient outcomes. CaHA may tighten and volumize the augmented tissue both with volumetric filling (CMC gel) and the induction of collagen and elastin synthesis. The initial volumizing effect is instantly supplied with the CMC gel and diluent, whereas the biostimulatory volumiziation is gradual. These results improve over time as biostimulation peaks. Additionally, CaHA can be used to augment previous surgical procedures such as BBLs, fat transfers, or cellulite treatments using collagenases. For example, postoperative complications following surgical BBLs may include asymmetries, cellulite caused by fat transfer, stretch marks from increasing volume too quickly, and correcting uneven surfaces caused by traumatic insertion or fat grafting. CaHA may be used combinatorially with implant and with fat transfer procedures. In addition, it is generally advisable for patients to wait anywhere between 4-6 weeks for any additional procedures performed in the same anatomy.

One of the most desirable features of the BVIBBL is that as the process of neocollagenesis and neoelastogenesis initiates, the long-term clinical outcomes are improved. After around four to six weeks, one may start seeing significant tissue lift, tissue tightening, skin texture improvement, decrease in cellulite, and minimization of stretch marks. HDR CaHA will maintain anywhere between 18-24 months, creating a long-term scaffolding for endogenous fibroblasts. The BVIBBL was created as a single treatment for gluteal augmentation, though patients may request follow-up treatments to achieve altered aesthetic outcomes. To this end, follow-up treatments can occur as soon as four to six weeks after the initial injection, though no dosing interval is suggested.

**Figure 5:**
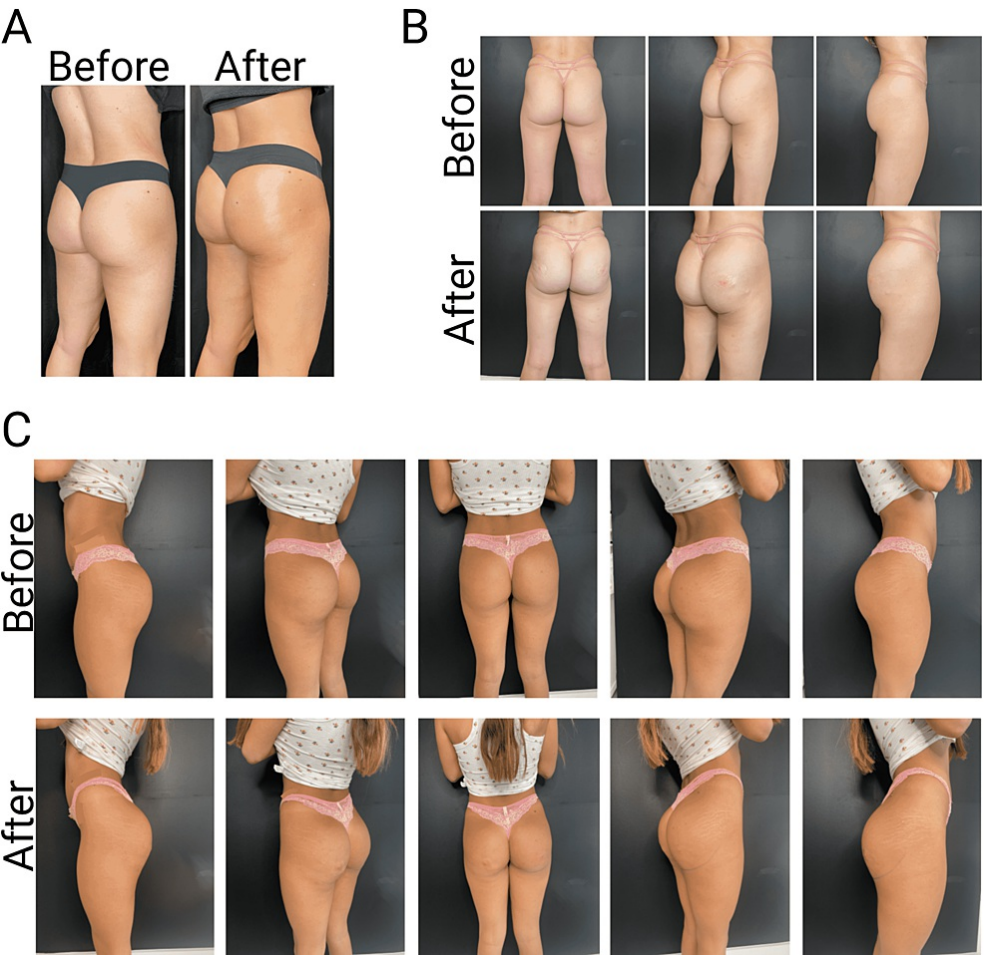
Before and after injection of 10 syringes per side (20 total) in three patients (A) Female patient age 26, desired hip dips and projection; (B) Female patient age 24, desired shape hip dips; (C) Female patient age 27, desired shape full volume and projection.

Managing patient expectations is very important so that the patient satisfaction standard is met. At the end of the procedure, patients should see before and after pictures as well as measurements to confirm an increase in size and visual corrections occurred (Figure 5). Questions do arise about potential complications that can occur, and addressing these concerns vary from vascular occlusions (which generally do not occur with such a large bore cannula or with such diluted product), to bruising (which is common with any aesthetic procedure). Although a very low percentage of patients develop hematomas, it is possible. Granulomas or bumps or lumps are a main concern for the patient, but the product will settle within the first 24-48 hours. Filler migration is not a concern because of the high viscoelastic properties of even HDR CaHA.

Complications associated with the BVIBBL are minimal. Expected adverse events (AEs) with HDR CaHA commonly consist of bruising, swelling, hyperpigmentation at the insertion point, soreness or pain at the injection site, and possible injection site infection. To date, no study has sought to observe these AE rates in patients undergoing gluteal augmentation. While these AEs are easily managed by a variety of minor interventions (oral non-steroidal anti-inflammatory drugs, warm compression, etc.). Although not likely, these complications can arise, and it is necessary for HCPs to have protocols in place to manage them. A variety of protocols exist for the management of serious and non-serious AEs using HDR CaHA [25].

## Conclusions

This technical report summarizes an algorithmic, patient-specific method, the Bella Vida Instant BBL™, for bilateral gluteal augmentation using a hyperdiluted biostimulatory CaHA filler. HDR CaHA drives clinical outcomes via the immediate volumization offered by CMC gel and the long-term and gradual biostimulation of type 1 and type 3 collagen and elastin. These results, when paired with the BVIBBL technique, result in a volumized, non-cellulitic, symmetrical, and natural-looking gluteal augmentation that is practical for implementation in a variety of practices. The practice of hyperdilution increases the tissue volume undergoing biostimulation, reduces the risk of nodule formation and vascular occlusion, and makes volumization instant. Four specific clinical outcomes are described in this report, with detailed overviews of the pre-, intra-, and post-procedural steps. Overall, the BVIBBL may meet the emerging demand for minimally invasive gluteal augmentation procedures.

## References

[1] Wang JV, Akintilo L, Geronemus RG (2020). Growth of cosmetic procedures in millennials: A 4.5-year clinical review. J Cosmet Dermatol.

[2] Sobanko JF, Dai J, Gelfand JM, Sarwer DB, Percec I (2018). Prospective cohort study investigating changes in body image, quality of life, and self-esteem following minimally invasive cosmetic procedures. Dermatol Surg.

[3] Hopkins ZH, Moreno C, Secrest AM (2020). Influence of social media on cosmetic procedure interest. J Clin Aesthet Dermatol.

[4] Nestor MS, Newburger J, Zarraga MB (2013). Body contouring using 635-nm low level laser therapy. Semin Cutan Med Surg.

[5] Weiss RA (2013). Noninvasive radio frequency for skin tightening and body contouring. Semin Cutan Med Surg.

[6] Nassab R (2015). The evidence behind noninvasive body contouring devices. Aesthet Surg J.

[7] Lorenc ZP, Black JM, Cheung JS (2022). Skin tightening with hyperdilute CaHA: dilution practices and practical guidance for clinical practice. Aesthet Surg J.

[8] Shridharani SM, Tisch GM, Ebersole TG, Moak TN, Edwartz C (2021). Clinical experience of poly-L-lactic acid injections for body contouring treatment. J Cosmet Dermatol.

[9] Corduff N, Chen JF, Chen YH (2021). Pan-Asian consensus on calcium hydroxyapatite for skin biostimulation, contouring, and combination treatments. J Clin Aesthet Dermatol.

[10] de Almeida AT, Figueredo V, da Cunha AL (2019). Consensus recommendations for the use of hyperdiluted calcium hydroxyapatite (Radiesse) as a face and body biostimulatory agent. Plast Reconstr Surg Glob Open.

[11] Logas C, Kosche C, Perez M, Martinez-Diaz GJ (2018). Biostimulatory injectables for the treatment of cellulite and gluteal enhancement. Plast Reconstr Surg.

[12] Yutskovskaya YA, Kogan EA (2017). Improved neocollagenesis and skin mechanical properties after injection of diluted calcium hydroxylapatite in the neck and décolletage: a pilot study. J. Drugs Dermatol.

[13] Yutskovskaya YA, Sergeeva AD, Kogan EA (2020). Combination of calcium hydroxylapatite diluted with normal saline and microfocused ultrasound with visualization for skin tightening. J Drugs Dermatol.

[14] Kim J (2019). Multilayered injection of calcium hydroxylapatite filler on ischial soft tissue to rejuvenate the previous phase of chronic sitting pressure sore. Clin Cosmet Investig Dermatol.

[15] Goldie K, Peeters W, Alghoul M (2018). Global consensus guidelines for the injection of diluted and hyperdiluted calcium hydroxylapatite for skin tightening. Dermatol Surg.

[16] Sinno S, Chang JB, Brownstone ND, Saadeh PB, Wall S Jr (2016). Determining the safety and efficacy of gluteal augmentation: a systematic review of outcomes and complications. Plast Reconstr Surg.

[17] Mendieta CG (2006). Intramuscular gluteal augmentation technique. Clin Plast Surg.

[18] Harrison D, Selvaggi G (2007). Gluteal augmentation surgery: indications and surgical management. J Plast Reconstr Aesthet Surg.

[19] Cardenas Restrepo JC, Muñoz Ahmed JA (2002). Large-volume lipoinjection for gluteal augmentation. Aesthet Surg J.

[20] Tijerina JD, Morrison SD, Nolan IT, Vail DG, Lee GK, Nazerali R (2020). Analysis and interpretation of Google Trends data on public interest in cosmetic body procedures. Aesthet Surg J.

[21] Tijerina JD, Morrison SD, Nolan IT, Vail DG, Nazerali R, Lee GK (2019). Google Trends as a tool for evaluating public interest in facial cosmetic procedures. Aesthet Surg J.

[22] Tijerina JD, Morrison SD, Nolan IT, Parham MJ, Nazerali R (2020). Predicting public interest in nonsurgical cosmetic procedures using Google Trends. Aesthet Surg J.

[23] Tijerina JD, Morrison SD, Vail DG, Lee GK, Nazerali R (2019). The utility of Google Trends data for analyzing public interest in breast procedures. Ann Plast Surg.

[24] McCarthy AD, McGoldrick DJ, Holubeck PA, Cohoes C, Bilek LD (2021). Social data: an underutilized metric for determining participation in COVID-19 vaccinations. Cureus.

[25] van Loghem J, Funt D, Pavicic T (2020). Managing intravascular complications following treatment with calcium hydroxylapatite: an expert consensus. J Cosmet Dermatol.

