# Correction: Bilateral Gluteal Augmentation With Hyperdilute Calcium Hydroxylapatite Microspheres Performed Using the Bella Vida Instant Brazilian Butt Lift (BBL)™

**DOI:** 10.7759/cureus.c67

**Published:** 2022-06-27

**Authors:** Iani Silveira, Brigitte Martinez

**Affiliations:** 1 Dermatology, Bella Vida Aesthetics & Wellness, Miami, USA; 2 Nursing, Keiser University, Miami, USA

Upon publication, the Cureus editorial office noticed that a key financial relationship was not disclosed. The Financial Relationships section of the Ethics Statement and Conflict of Interest Disclosures has therefore been updated to include the following:

Iani Silveira declare(s) employment from Bella Vida Aesthetics. Iani Silveira is the owner of Bella Vida Aesthetics, which advertises the Bella Vida Instant Brazilian Butt Lift procedure as a paid service.

When contacted, the authors were in agreement that this relationship be disclosed via formal correction. The Cureus editorial office and the authors apologize that this oversight was not identified and addressed prior to publication.

